# Spatial differences and influence mechanisms of construction land development intensity in China, 2002–2020

**DOI:** 10.1038/s41598-023-36819-5

**Published:** 2023-07-10

**Authors:** Guanhai Gu, Bin Wu, Qichen Chen, Wenzhu Zhang, Rucheng Lu, Shengquan Lu, Xiaoling Feng, Wenhui Liao

**Affiliations:** 1 School of Natural Resources and Surveying and Mapping, Nanning Normal University, Nanning, 530100 China; 2 School of Law, Hunan University, Changsha, 410012 China

**Keywords:** Sustainability, Urban ecology

## Abstract

Construction land development intensity is a spatial mapping of modern urbanization level, which integrally reflects urban development strategy, land use efficiency, and population carrying intensity. This article analyzed the spatial and temporal evolution of construction land development intensity using panel data of 31 provincial administrative divisions in China from 2002 to 2020, with the application of the Theil index and spatial autocorrelation. To further investigate the relationship between human activities and land development, the article used geographic detectors to analyze the influencing mechanisms. The results showed that: (1) The average intensity of construction land development of Chinese provinces from 2002 to 2020 showed a trend of "steady increase, a short decline, and then a steady increase," and there were significant differences in the characteristics of construction land development intensity changes in different regions. (2) The regional differences in construction land development intensity between provinces showed a decreasing trend. There were uneven differences among regions, with more minor regional differences in Central, South, and North China but more significant differences in Northwest, East, Southwest, and Northeast China. (3) The spatial agglomeration of construction land development intensity in the region increased initially and then decreased during the study period. The overall pattern was "small agglomeration and large dispersion." (4) Economic development factors such as GDP per land, industrial structure, and fixed asset investment completion significantly affect land development intensity. The interaction between the factors was apparent, and the effect of “1 + 1 > 2” was produced. Based on the study's results, it is suggested that scientific regional development planning, guiding inter-provincial factor flow, and rational control of land development efforts are the key to promoting sustainable regional development.

## Introduction

Construction land development intensity is a comprehensive indicator of the degree of construction land saving and intensification and the activity of economic production, and a mapping of the relationship between land use structure and population and social economy under the level of modern urbanization. Since the reform and opening-up, with the rapid development of China’s economy and the continuous promotion of urbanization, land resources, as a carrier of human economic activities and production factors, have become a catalyst for the expansion of urban development. However, the disorderly urban expansion and the traditional rough development mode make land use inefficient, resulting in low construction land development intensity. In the new stage and situation of urban development, the 14th Five-Year Plan in China proposes to actively promote the development of a new type of urbanization and promote the high-quality development of China’s cities. How to balance improved land use and high-quality social economic development under the premise of scarce land resources has become the focus of government and academia. At the same time, it is a necessary choice to support the sound development of society^1^. However, China’s economic development is uneven across provinces, with significant heterogeneity in land development intensity, and the contradiction between land supply and demand is becoming increasingly prominent in many regions. There is an urgent need to increase the intensity of land development to alleviate the conflict, but too much intensity can seriously threaten food security, ecological safety, and sustainable urban development^2^. Therefore, controlling moderate construction land development intensity is an inevitable requirement to configure the scale of urban space and improve the efficiency of land use and the healthy development of the future regional economy. Accordingly, it is of practical significance to strengthen the research on the spatial and temporal evolution and driving mechanism of construction land development intensity, to reasonably control the land development boundary, optimize the land use structure, promote the organic unity of social, economic, and ecological benefits of land use, and promote the sustainable development of the region.

A review of the literature on construction land development intensity reveals that research has focused on three main areas. (1) Regarding the definition of the connotation of construction land development intensity, some studies have pointed out that the concept of construction land development intensity originated more from agricultural land, evolved from agricultural development, and gradually extended to be used to measure the efficiency of construction land use and other fields with the rapid development of urbanization^2–4^. From the perspective of "intensity," some scholars have revealed the complexity and multiplicity of land development and utilization intensity^5^, reflecting the degree of land development and utilization and its social economic, demographic, and ecological carrying intensity, comprehensively reflecting the cumulative degree and carrying density of land utilization, and mapping the modernization level of cities in terms of space^6–10^. It is also a combination of the conditions, breadth, and depth of land development and uses and the level of inputs and output returns^11–13^. (2) In terms of measuring and evaluating construction land development intensity. There are two main approaches in terms of indicator systems: one is a single indicator measure, where construction land development intensity can be recognized as a process of land conversion from non-built-up areas to built-up areas^3,4^, as well as a measure of the ratio of built-up land to the unit area, building density and volume ratio^14,15^. The second is the composite indicator measure, which measures the intensity of land development from several indicators such as land use structure, land input and output, land conservation and intensification, construction land density, and population carrying capacity^6,8,10,11,16^. The evaluation method of construction land development intensity is mainly based on panel data and spatial analysis model and combined with "3S" and spatial analysis technology to improve the evaluation accuracy. Comparing the two methods, the advantage of the single indicator measurement lies in its simplicity and ease of interpretation. However, it may oversimplify the meaning of construction land development intensity due to its limited number of indicators. On the other hand, the comprehensive index measure offers a more comprehensive approach and can reflect the development intensity of construction land from multiple dimensions, including the degree of intensive land use and economic production activities. (3) In terms of spatial and temporal changes in construction land development intensity and influencing factors. Many studies have employed remote sensing and GIS technologies to examine the development trajectory and attributes of construction land development intensity regarding urban land expansion, conversion, and change^17,18^. Additionally, these studies have also built models to uncover the underlying causes of affecting factors^19–21^. Construction land development intensity is complex and results from a multifactorial combination. Some scholars have launched studies on the impact of construction land development intensity changes on the ecological environment, ecosystem service functions, and the interrelationship with biodiversity^22,23^, complementing the studies on the interrelationship between land use and other factors.

In general, with the support of RS, GIS, and other technologies, the analysis of land development and intensity has yielded rich results. Based on previous literature, the research primarily focuses on the evaluation method of construction land development intensity. Subsequently, it delves into the analysis of spatiotemporal changes in land use intensity and the impact of various elements on land use intensity. However, the regional variability of the spatial variation of the development intensity of construction land and the long-term continuous evolution of its potential impact mechanism still needed to be explored in depth. Different geographic spaces exhibit distinct regional characteristics in land use, and the interplay between the natural environment and social economic factors also manifest notable spatial differences in local development across various regions. Therefore, directing attention towards regional disparities can effectively uncover the patterns of construction land development intensity and shed light on the driving mechanisms involved. Currently, China is in a rapid stage of urbanization, which has led to increasingly evident regional development differences due to varying resource endowments in each region. Moreover, in some regions, there is a tendency to blindly pursue land development without sufficient emphasis on improving land use efficiency. This approach contradicts the principles of new urbanization and high-quality development^24^. Therefore, this study systematically collects panel data from 31 provinces in China from 2002 to 2020, using the construction land development intensity measurement model, Theil index, and spatial autocorrelation model. The analysis identifies the characteristics of the inter-provincial spatial and temporal patterns of construction land development intensity and reveals the dominant factors and driving mechanisms behind their spatial and temporal divergence through a geographic detector model. The results of this study have important implications for clarifying the direction of urban development, improving the quality of regional development, and avoiding the haphazard growth of urban space.

## Results

### Spatial and temporal evolution characteristics of construction land development intensity

#### Spatial and temporal patterns of construction land development intensity

**Figure 1:**
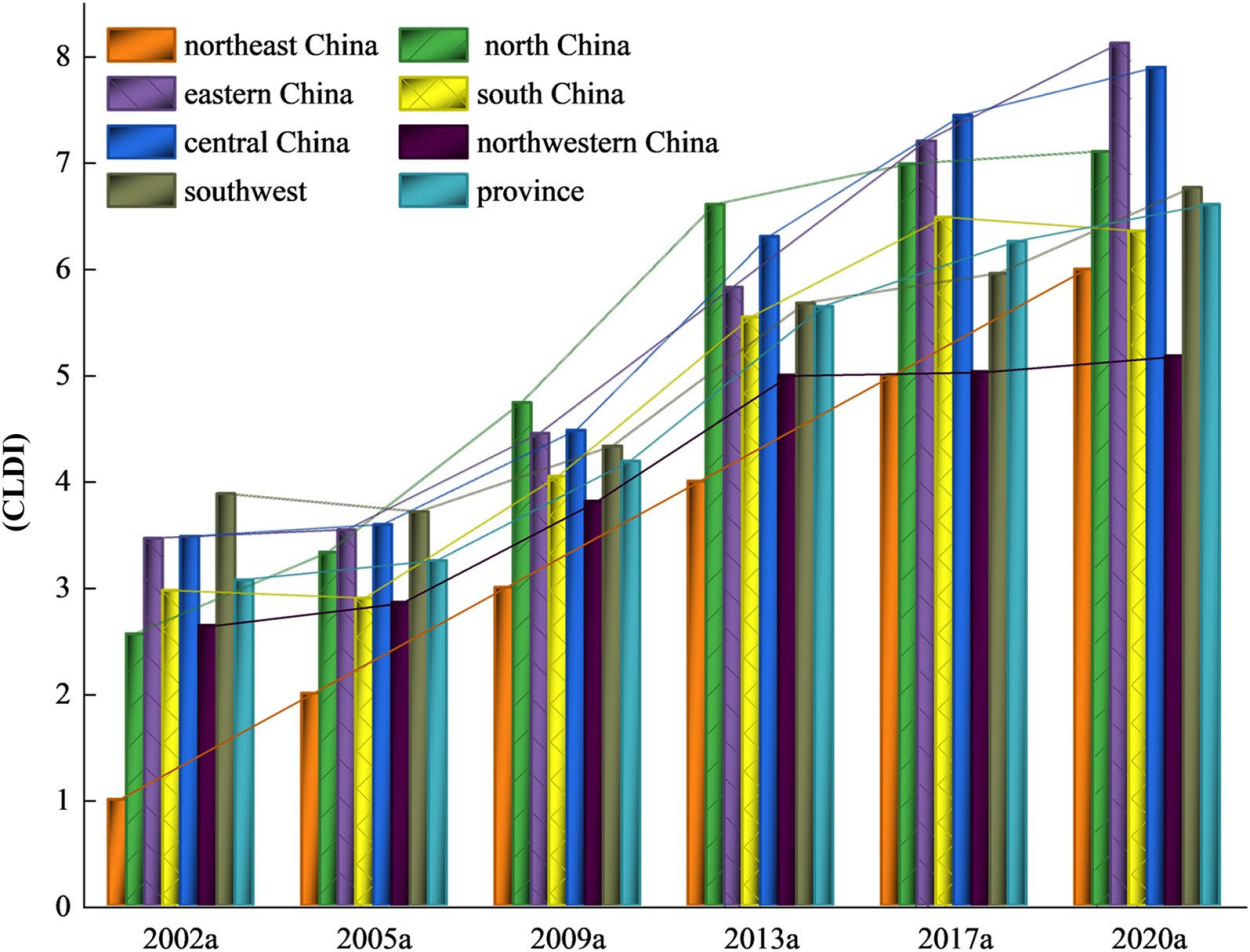
Construction land development intensity changes in regional development intensity changes from 2002 to 2020.

The level of construction land development intensity is measured based on the degree of construction land intensity, population per unit of land, and social economic carrying capacity. We analyze the change in construction land development intensity in each region of China and the trend of the mean value of construction land development intensity in China, Northeast China, North China, Central China, East China, South China, Southwest China, and Northwest China was plotted (Fig. 1). Overall, China’s construction land development intensity has been on an upward trend from 2002 to 2020, with the average intensity increasing from 3.07 in 2002 to 6.61 in 2020. There have been more obvious phase characteristics in the growth rate of the average intensity of construction land development. The growth rate showed a steady increase from 2002 to 2013, which coincided with the rapid social economic development of China and the significant improvement of people’s living standards, and also drove the process of land development and urban expansion. The growth rate began to show a significant downward trend after 2013, mainly due to the global economic downturn. It increased downward pressure on the domestic economy. In 2017 the country began to pay attention to expanding domestic demand to promote development, economic structure transformation, and upgrading, bringing a direct impact on land development and land output, resulting in the growth rate of land development intensity turned up; In 2020, when the COVID-19 pandemic hit the world, the land development intensity dropped to 6.61. By region, the overall level is higher in Central China, East China, and North China, followed by Southwest China, South China, and Northwest China, and lowest in Northeast China. Each region’s average intensity of construction land development curves showed different trends during the study period, among which Central and Eastern China showed a stable upward trend during the study period except for 2019–2020a when a decline was observed due to the impact of COVID-19. North China underwent the same broad changes as Central and East China, but experienced a significant decline from 2009 to 2013. Southwest and South China maintained a stable low upward trend before 2008, with fluctuations in the shape of an "S" after that. The Northwest region showed a steady upward trend until 2013, followed by fluctuations around a stable value. The Northeast region, on the other hand, displayed a linear increase in the intensity of construction land development throughout the study period.

#### Analysis of regional differences in construction land development intensity

Matlab 2017b software is used to calculate the Theil index of construction land development intensity for 31 provincial administrative regions in China from 2002 to 2020 and grouped by geographical subdivisions to analyze the characteristics of land use and development intensity and the differences between provinces and regions. The results of the overall Theil index, intra-group Theil index, inter-group Theil index, and Theil index by region are shown in Table 1.

**Table 1:** Theil index results of construction land development intensity from 2002 to 2020.

Year	Theil Index	Group inner index	Theil index between groups	Northeast China	North China	Eastern China	South China	Central China	Northwest China	Southwest China
2002	0.0551	0.0381	0.0170	0.0100	0.0553	0.0493	0.0063	0.0206	0.0261	0.0604
2003	0.0479	0.0343	0.0137	0.0127	0.0577	0.0370	0.0087	0.0183	0.0366	0.0482
2004	0.0402	0.0296	0.0106	0.0273	0.0243	0.0269	0.0133	0.0211	0.0351	0.0462
2005	0.0425	0.0357	0.0068	0.0284	0.0262	0.0354	0.0579	0.0175	0.0452	0.0398
2006	0.0390	0.0319	0.0071	0.0241	0.0184	0.0299	0.0664	0.0120	0.0493	0.0298
2007	0.0312	0.0256	0.0057	0.0232	0.0136	0.0268	0.0679	0.0077	0.0317	0.0191
2008	0.0354	0.0287	0.0067	0.0370	0.0120	0.0274	0.0499	0.0143	0.0540	0.0171
2009	0.0323	0.0264	0.0059	0.0267	0.0063	0.0291	0.0068	0.0085	0.0806	0.0147
2010	0.0379	0.0353	0.0026	0.0315	0.0754	0.0255	0.0133	0.0143	0.0669	0.0155
2011	0.0319	0.0276	0.0043	0.0294	0.0112	0.0262	0.0166	0.0129	0.0770	0.0139
2012	0.0335	0.0291	0.0045	0.0227	0.0128	0.0279	0.0086	0.0150	0.0767	0.0259
2013	0.0330	0.0263	0.0067	0.0215	0.0141	0.0265	0.0169	0.0117	0.0633	0.0234
2014	0.0328	0.0257	0.0071	0.0155	0.0145	0.0260	0.0046	0.0208	0.0665	0.0230
2015	0.0344	0.0231	0.0113	0.0142	0.0045	0.0236	0.0277	0.0090	0.0573	0.0252
2016	0.0364	0.0194	0.0170	0.0178	0.0028	0.0106	0.0055	0.0091	0.0548	0.0381
2017	0.0310	0.0155	0.0155	0.0230	0.0113	0.0098	0.0008	0.0126	0.0491	0.0082
2018	0.0356	0.0189	0.0167	0.0317	0.0108	0.0136	0.0100	0.0107	0.0511	0.0128
2019	0.0453	0.0209	0.0244	0.0338	0.0244	0.0206	0.0071	0.0070	0.0419	0.0138
2020	0.0462	0.0226	0.0236	0.0497	0.0284	0.0200	0.0134	0.0095	0.0408	0.0095

The Theil index of inter-provincial construction land development intensity in China tends to decrease from 2002 to 2020, and regional differences tend to narrow. From 2002 to 2016, the Theil index of construction land development intensity showed a decreasing trend, from 0.0551 to 0.0364; after 2017, the Theil index started to increase, from 0.0310 to 0.0462 year by year, but lower than in 2002. The intra-group Theil index shows a decreasing trend, and the inter-group Theil index decreases and then increases, indicating that the overall difference in construction land development intensity within provinces decreases while the difference between provinces increases. At the beginning of the twenty-first century, China’s economy began to enter a stage of rapid development. Due to the regional economic differences caused by the uneven allocation of resource endowments and production factors in each region, vigorously promoting coordinated regional development has been gradually reduced, but the "siphon effect" between regions still makes the inter-provincial gap widen.

From the perspective of each region, the Theil Index of construction land development intensity in Central China, South China, and North China is relatively small, except for South China and North China, where the values of the Theil Index fluctuated more than 0.05 in 2006a and 2010a, respectively. It shows that the differences in construction land development intensity in the three regions are insignificant, and the heterogeneity of construction land development intensity in the provinces is balanced. The Theil index of construction land development intensity in Northwest China, East China, Southwest China, and Northeast China is more extensive, among which the trend of the Theil index of land use intensity in Southwest China and East China is "decreasing, fluctuating, rebounding and then decreasing." The overall Theil index value decreases from 0.0604 and 0.0493 in the base year to 0.0095 and 0.0200 in 2020, respectively, which shows that the regional differences in construction land development intensity between the two regions are decreasing. The changes in the Theil index of construction land development intensity in the northeast and northwest regions show a continuous fluctuating upward trend, and the fluctuation of the northwest region is more significant than that of the northeast region. Generally, the Theil index of the two regions has an upward trend, indicating that the construction land development intensity gap between the provinces and regions has increased.

### Spatial autocorrelation analysis

The Geode software calculated China’s global Moran index of construction land development intensity from 2002 to 2020 (Table 2). If the index is more significant than zero, it indicates that the variables have a positive spatial correlation; if the index is less than zero, there is a negative spatial correlation between cells, and if the index is zero, it means there is no spatial correlation. During the study period, the Moran index of construction land development intensity was positive in all years except for 2005 and 2008–2011. In terms of significance tests, only after 2015 passed a test of less than 5% or 1% with an index greater than 0, indicating a positive spatial correlation of construction land development intensity in the study area after 2015. Moran index values fluctuated and changed during the study period, and the Moran index did not pass the significance level test from 2002 to 2014, indicating that the spatial correlation of construction land development intensity was not significant then. The Moran index value of 0.127 in 2015 was significant at the 5% level and continued to rise to 0.251 by 2018, during which the spatial correlation of construction land development intensity tended to strengthen. The Moran index of construction land development intensity in the study area continues to decline from 2019 to 2020 but still passes the significance test, indicating a weakening trend of spatial aggregation.

**Table 2:** Moran index values of construction land development intensity from 2002 to 2020.

Year	Moran’s I	Z-statistic	P-value	Year	Moran’s I	Z-statistic	P-value
2002	0.079	1.467	0.142	2012	0.007	0.520	0.603
2003	0.053	1.111	0.267	2013	0.050	1.061	0.289
2004	0.051	1.099	0.272	2014	0.047	1.033	0.302
2005	− 0.004	0.376	0.707	2015	0.127	2.046	0.041
2006	0.009	0.539	0.590	2016	0.202	3.026	0.002
2007	0.013	0.590	0.555	2017	0.229	3.365	0.001
2008	− 0.001	0.409	0.683	2018	0.251	3.639	0.001
2009	− 0.003	0.393	0.694	2019	0.223	3.303	0.001
2010	− 0.143	− 1.406	0.160	2020	0.205	3.073	0.002
2011	− 0.038	− 0.063	0.950				

**Figure 2:**
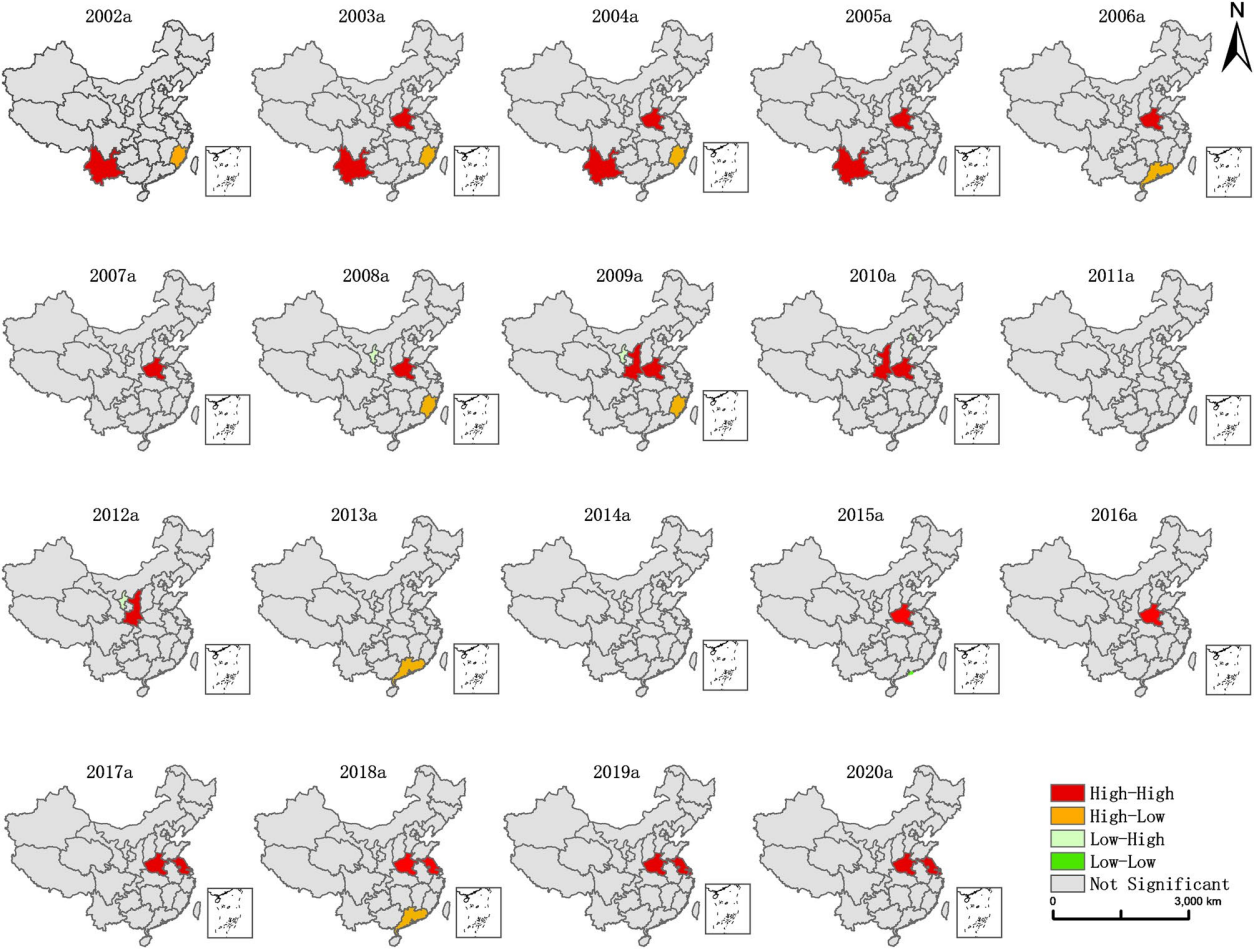
LISA chart of construction land development intensity from 2002 to 2020. The map was created using Esri's ArcGIS 10.8 desktop GIS software. The base map is based on the standard map with review number GS (2020) 4619 downloaded from the website of China Ministry of Natural Resources standard map service.

Global spatial autocorrelation can only reflect the average degree of the unit and the surrounding units. To reveal the local spatial clustering of construction land development intensity in the 31 provincial administrative regions, four clustering patterns: high-high clustering (HH), high-low clustering (HL), low–high clustering (LH), and low-low clustering (LL) will be used further to analyze the local spatial autocorrelation of the study area. Furthermore, based on this, we draw the spatial clustering map of construction land development intensity in 31 provinces from 2002 to 2020a (Fig. 2).

From a comprehensive point of view, the spatial aggregation of construction land development intensity from 2002 to 2020 is not high, and the overall pattern of local correlation shows a "small aggregation and large dispersion." HH and HL dominate the clustering, and the distribution is concentrated in Southwest China, Central China, and East China.

From the regional spatial clustering in the study period, the clustering pattern from 2002 to 2006 was mainly of HH and HL type, with the HH type concentrated in Henan and Yunnan provinces. Since Henan Province has the highest population, the construction land area and the output value of secondary and tertiary industries are in the middle and upper levels, but the total area is only in the 18th position. The construction land area and the output value of secondary and tertiary industries in Yunnan Province are in the middle and lower levels. However, its population and total area are in the middle level, and the degree of land development is more appropriate to the economic carrying capacity of the population. Thus, the intensity of land development is higher in both provinces. HL type mainly occurs in Fujian Province and Guangdong Province. Three types emerged from 2007 to 2011: HH, LH, and HL types, and Henan Province in 2007a still maintained the HH status in the first four periods, and all 31 provinces were spatially aggregated insignificantly by 2011. LH and HH aggregation appeared in Ningxia and Shaanxi provinces in 2012, after which the spatial agglomeration of construction land development intensity in each province began to weaken. In 2018, HL aggregation appeared in Guangdong Province, and HH aggregation was maintained in Henan Province and Shanghai, Jiangsu Province from 2017 to 2020, closely related to the region’s economic development and population aggregation. It can be seen that from 2002 to 2020, most of the provinces did not have significant spatial aggregation, and the overall pattern shows a "small aggregation, large dispersion" state. Therefore, the provinces should promote factor interconnection and coordinated regional development. At the same time, it should strictly control the scale of construction land use, encourage the transformation of industrial structure and economic development, take a new type of urbanization development path, improve high-quality land use and sustainable development, increase inter-provincial linkage development, and promote regional high-low gathering.

### Analysis of the drivers of change in construction land development intensity in China

Referring to the studies of related scholars^2,13,25^, we found that social economy, natural environment, and national policies can influence construction land development intensity. Based on the availability of data and the region’s characteristics, we selected the following 16 indicators as influencing factors for economic development, social investment, and infrastructure construction. They include average local GDP (X1), the proportion of secondary and tertiary industries (X2), total retail sales of consumer goods (X3), average local population (X4), international tourism income (X5), and average local food production (X6), which indicate the regional population distribution, industrial structure, and economic development. New construction land (X7), completed fixed asset investment in municipal utility construction (X8), local fiscal expenditure (X9), general budget revenue (X10), road network density (X11), and total water supply per land (X12), which indicate the scale of asset investment and public facility construction capacity of the region. Green coverage area (X13), sewage treatment rate (X14), number of primary and secondary school students (X15), and number of health institutions (X16), which indicate the level of infrastructure service construction and green development in the area.

#### Factor detection results

**Figure 3:**
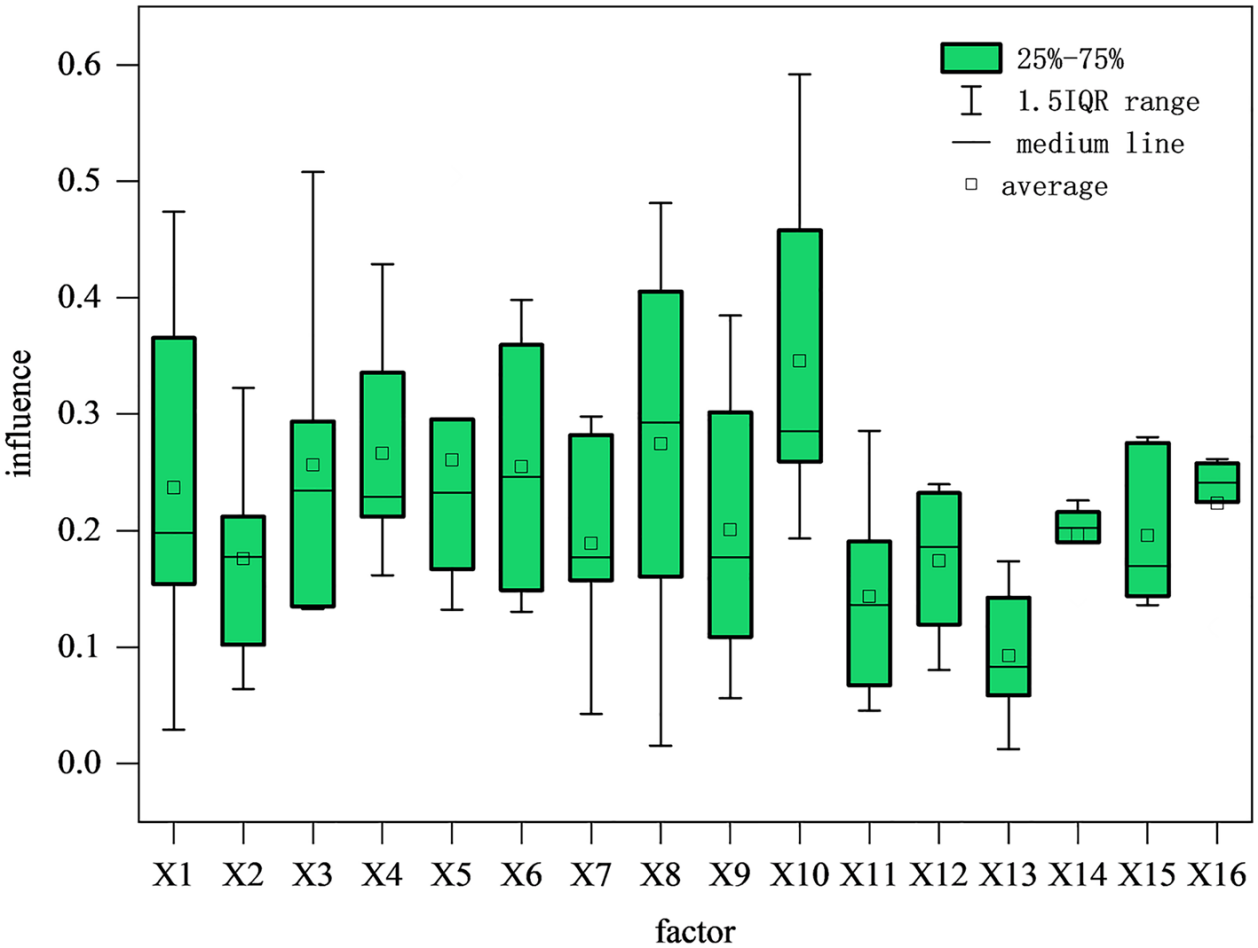
Changes in the influence of inter-provincial drivers of construction land development intensity in China from 2002 to 2020.

The spatial and temporal evolution of interprovincial construction land development intensity and its spatial autocorrelation show significant differences in its spatial differentiation due to the combined impact of multiple factors. The analysis of the variation in the driving factors of construction land development intensity among provinces in China (Fig. 3) reveals that the influence of construction land development intensity varied in the study area from 2002 to 2020, with a mean value of q ranging between 0.15 and 0.25. Overall, economic development-type factors play a dominant role in the spatial pattern of inter-provincial construction land development intensity since the transformation and upgrading of industrial structure, production factors, and population gathering cannot be separated from the support of land resources. At the same time, construction land development intensity, to some extent, also reflects the process of economic development. Social investment-based drivers play an essential role, and the scale of capital investment can reflect the regional economic development potential to some extent. With the vigorous promotion of supply-side structural reform in recent years, the investment structure has been optimized, and the quality and efficiency of investment have continued to improve, with an increasingly significant impact on the spatial pattern of construction land development intensity. The essential constructive factor mainly influences construction land development intensity through the expansion of construction land scale, and its influence on the spatial pattern of construction land development intensity is relatively weak.

#### Factor interaction detection results

**Figure 4:**
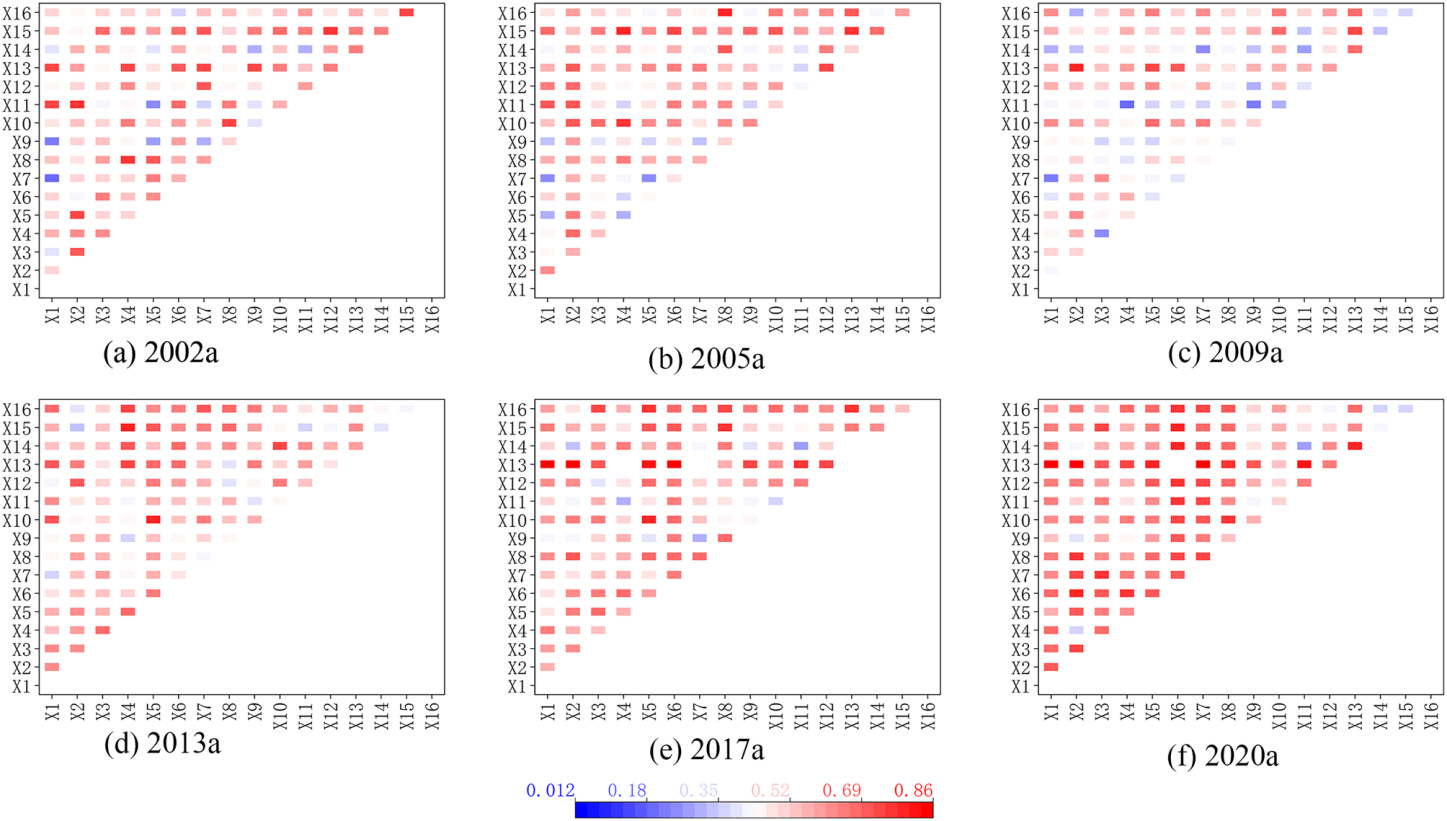
Interaction of Interprovincial Construction land development intensity Drivers in China, 2002–2020.

The analysis of the interaction detection of the influencing factors (Fig. 4) revealed that each influencing factor interacts with the evolution of the spatial pattern of interprovincial construction land development intensity. The different influencing factors contribute to the change of construction land development intensity after two-by-two interactions, and the results all show different degrees of two-factor enhancement or non-linear enhancement. It shows that the spatial differentiation of inter-provincial construction land development intensity is not only influenced by individual factors, but the combined effect of multiple factors such as economic development, social investment, and infrastructure construction drives the variability of the spatial pattern of provincial construction land development intensity. The interaction of land-average GDP with other factors is the strongest, and the increase of its post-interaction explanatory power is the highest, followed by the interaction of social development factors such as secondary and tertiary industries, total Theil sales of social consumer goods, and the completion of fixed asset investment with their remaining factors. The influence of road network density, the number of primary and secondary school students, the number of health institutions, the area covered by greenery, etc., and the interaction of population and industrial structure is non-linearly increasing, indicating that with the development of society, strengthening the construction of infrastructures such as medical care, education, and transportation, improving the capacity and level of urbanization construction has gradually significant influence on the spatial pattern of land development intensity. In general, the size of the population not only directly affects the demand for land for living and production, such as residential land and industrial land but also gives rise to the demand for public services, commercial land, infrastructure, and other supporting facilities. At the same time, the optimization and upgrading of the industrial structure provide more employment opportunities for the society and increases the concentration of the population, which promotes the change of urban land structure and spatial structure and increases the demand for land for residential land, public facilities, green space, and transportation facilities. With rapid urbanization and industrialization, local governments aim to spur land development by attracting investment and establishing industrial parks. Essential infrastructure, including transportation, education, healthcare, and cultural facilities, also plays a crucial role in boosting land development. Investing in infrastructure will drive the growth of economic development zones and high-tech industrial parks, providing financial support for land development and sustained social economicgrowth^26^.

## Conclusion

Based on panel data from 31 Chinese provinces and cities, this paper comprehensively measures the level of construction land development intensity from 2002 to 2020, analyzes the characteristics of spatial and temporal patterns of construction land development intensity by combining the Theil index and spatial autocorrelation analysis, and identifies the main influencing factors and interaction relationships through a geographic probe model, and draws the following conclusions.

The growth rate of the average inter-provincial construction land development intensity from 2002 to 2020 was 115.31%. The trend showed an overall upward movement and distinct stage characteristics, which can be described as going through three phases: a steady increase, followed by a brief decrease, then a steady increase again. The construction land development intensity in different regions shows different change characteristics, among which the change trends in Central China, East China, and North China are more similar, showing a stable upward trend in general, with a decline in individual years during the period but a quicker rebound. Southwest China, South China, and Northwest China are rising steadily and then changing with "S" shaped fluctuations, and the overall intensity is increasing. The intensity of land development in the northeast region fluctuates wildly, with a trend of "linear rise, then arching increase, then inverted V-shaped change."

During the research period, the overall Theil index value change rate of construction land development intensity at the provincial level in China was -16.15%, and regional differences developed towards a decrease. In terms of Theil index between groups and within groups, the change rates were − 40.68% and 38.82% respectively. The difference in land development intensity within the province showed a declining trend, while the inter-provincial difference showed an upward trend. There is an imbalance in the Theil index across regions, with more minor regional differences distributed in Central China, South China, and North China, while other regions have more significant variability. Regarding the spatial correlation of construction land development intensity, the spatial agglomeration shows a trend of strengthening first and then decreasing from 2002 to 2020. The overall pattern is "small agglomeration, large dispersion," and the spatial correlation is insignificant in most provinces and cities.

The spatial pattern of inter-provincial construction land development intensity is more strongly driven by the superposition of influencing factors, and the effect of “1 + 1 > 2” is produced. The spatial heterogeneity of inter-provincial construction land development intensity has become more and more evident since the twenty-first century, and factors such as GDP, industrial structure, education, and medical level, local financial revenue and expenditure, transportation construction level, and social infrastructure construction have a significant impact on the spatial pattern of construction land development intensity. The interaction of driving factors has a promoting effect on the spatial pattern change of construction land development intensity, with the power of interaction generally improving between each factor. The impact strength is mostly between 0.52 and 0.69, showing a synergistic enhancement or non-linear increase.

## Discussion

The results of the Theil index and spatial autocorrelation analysis show that the overall intensity of construction land development in Chinese provinces increased during the study period with significant regional and temporal variability, indicating uneven development. However, over time, the construction land development intensity between provinces showed a trend towards narrowing, which aligns with the findings of other research^9,13,27^. Currently, land revenue remains a significant source of fiscal revenue and a driving force for development in many regions. The future land use structure in China is expected to be characterized by the expansion of urban construction land areas at different spatial scales while still facing the challenges of unbalanced and insufficient development^28^. To promote healthy and sustainable regional development, the following suggestions are made from the perspective of the intensity of construction land development:

1. To create a scientifically sound regional development plan, it is necessary to consider various factors such as the extent of regional construction land preservation and intensification, the industrial structure, and the population's carrying capacity. The level of construction land development intensity varies significantly across provinces and regions in China, and various strategies are employed to address these differences. In areas with excessive intensity, measures are taken to limit land supply, while in areas with moderate intensity, the land structure is optimized. In cities with lower intensity, efforts are made to improve the construction level^29^. When formulating a regional land development plan, consideration should be given to the differences in regional development, urban scale, and industrial economy, and differentiated control strategies should be adopted to control the intensity of urban land development within a reasonable range.
2. To ensure healthy growth of regional construction land development, we must encourage the efficient flow of production factors between regions and prevent regional development from facing the " Matthew Effect". China's provinces exhibit varying advantages in areas such as economic development, social investment, infrastructure, and ecology. Regions need to break down trade barriers and reduce local protectionism, eliminate institutional barriers that hinder the flow of productive services, and enhance the complementarity of regional industrial structures to promote synergistic regional development^30^. The results of geographic research indicate that economic development and social investment have a significant impact on the development intensity of construction land in China's provinces. Hence, policymakers should develop rules to promote inter-provincial complementarity of these advantageous factors and drive comprehensive city development.
3. To promote a new development pattern and ecological civilization construction, it is crucial to achieve rational land development and enhance regional quality. Localities should increase the efficiency of agricultural land use, adopt an environmentally friendly land development model to increase land supply, find new development sources to meet the high demand for construction land, and maintain a balance between land development and supply for the region as a whole.

## Research methods

### Study area and data sources

**Figure 5:**
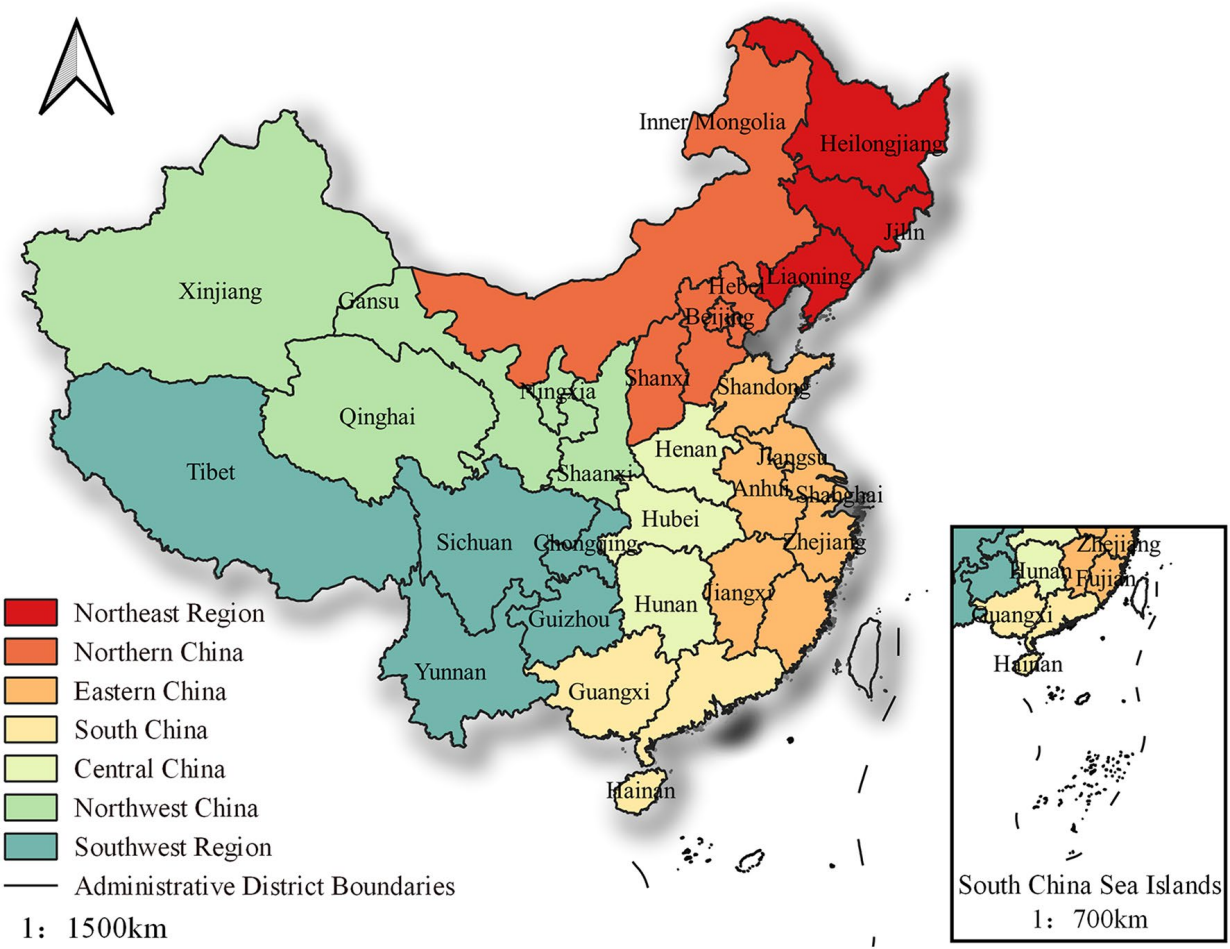
Study area map. The map was created using Esri's ArcGIS 10.8 desktop GIS software. The base map is based on the standard map with review number GS (2020) 4619 downloaded from the website of China Ministry of Natural Resources standard map service.

The study area of this paper encompasses 31 provincial administrative regions in mainland China, excluding Hong Kong, Macao, and Taiwan. To analyze the evolution of construction land development intensity in China’s provinces, they are grouped into seven regions based on geographical location and the natural environment: northeast, north, central, east, south, southwest, and northwest China (Fig. 5)^24^. The study period spans from 2002 to 2020. The panel data was mainly sourced from the China Statistical Yearbook, China Rural Statistical Yearbook, provincial and municipal statistical yearbooks, and the official website of the National Bureau of Statistics.

### Construction land development intensity index model

The construction land development intensity (CLDI) is a comprehensive index that reflects the level of development and utilization of construction land in a specific region, including the carrying capacity of the population and social economic factors. It is characterized by the construction land density, population carrying capacity, and land output intensity, providing a comprehensive evaluation of the region^8,31^. The equation for CLDI can be expressed as follows:

1 $$CLDI = \alpha CLUA + \beta PCC + \lambda OIL$$

where: *CLDI* represents construction land development intensity, *CLUA* represents construction land density (ratio of the regional construction area to total regional area), *PCC* represents population carrying capacity (ratio of the total population to construction land area), *OIL* represents land output intensity (ratio of secondary and tertiary industry value to construction land area), and $\alpha$, $\beta$, and $\lambda$ represent their respective weights, which are taken as 0.4, 0.3, and 0.3 in the paper.

#### Theil index

The Theil index is widely used to describe individual differences, structural differences, and regional differences, and this paper uses the Theil index to analyze the variability in the spatial and temporal evolution of construction land development intensity in China^32^, whose expressions are:

2 $$T = \frac{1}{n}\sum\limits_{i = 1}^{n} {\frac{{Y_{i} }}{{\overline{Y} }}} \log \left( {\frac{{Y_{i} }}{Y}} \right)$$

3 $$T_{b} = \sum\limits_{K = 1}^{K} {Y_{k} } \log \left( {\frac{{Y_{k} }}{{{{nk} /n}}}} \right)$$

4 $$T_{k} = \sum\limits_{K = 1}^{K} {Y_{k} } \left( {\sum\limits_{i = 1}^{n} {\frac{{Y_{i} }}{{Y_{k} }}} \log \left( {\frac{{{{Y_{i} }/{Y_{k} }}}}{{{1 / {nk}}}}} \right)} \right)$$

where: T represents the Theil index, $Y_{i}$ the construction land development intensity of 31 provinces, and the average intensity of construction land development. T_*b*_ is the gap within groups, T_*k*_ is the gap between groups, $Y_{i}$ is the construction land development intensity of province I, and $Y_{k}$ is the construction land development intensity of group k.

#### Spatial autocorrelation

Spatial autocorrelation includes global and local spatial autocorrelation, and the Moran index is used in this paper to test the global spatial autocorrelation of construction land development intensity in 31 provinces, and the Moran index is calculated as (5). The local Moran index examines the effect of agglomeration and dispersion of construction land development intensity in 31 provinces and analyzes the spatial autocorrelation of each unit with its neighboring units, and the Local Moran index formula is shown in (6)^33^.

5 $$Moran^{\prime}sI = \frac{{\sum\nolimits_{i = 1}^{n} {\sum\nolimits_{j = 1}^{n} {W_{ij} \left( {Y_{i} - \overline{Y} } \right)\left( {Y_{j} - \overline{Y} } \right)} } }}{{S^{2} \sum\nolimits_{n = 1}^{n} {\sum\nolimits_{j = 1}^{n} {W_{ij} } } }}$$

6 $$I_{i} = \frac{{n\left( {Y_{i} - \overline{Y} } \right)\sum\nolimits_{j = 1}^{n} {W_{ij} \left( {Y_{j} - \overline{Y} } \right)^{2} } }}{{\sum\nolimits_{n = 1}^{n} {\left( {Y_{i} - \overline{Y} } \right)} }}$$

where: $Y_{i}$ and $Y_{j}$ represent the construction land development intensity of province i and province j, respectively, and n is the number of variables. $W_{ij}$ is the spatial weight matrix and $I_{i}$ is the local Moran index.

#### Geographic detector

In this paper, we apply the factor detector and interaction detector to carry out the analysis to explore the influence of each influencing factor on the change of construction land development intensity and the intensity and type of multi-factor interaction^34^, which is expressed as:

7 $$p = 1 - \frac{{\sum\nolimits_{{{\text{i}} = 1}}^{N} {n_{i} \sigma_{i}^{2} } }}{{n\sigma^{2} }}$$

where: $p$ is the factor influence; $n_{i}$ and $n$ are the number of cells in the stratum and the whole region, respectively; $\sigma_{i}^{2}$, $\sigma^{2}$ are the y-value variances of the stratum and the whole region, respectively.

## Data Availability

The datasets used and/or analysed during the current study are available from the corresponding author upon reasonable request.
